# Supplementary material for: Membrane Fluidization Governs the Coordinated Heat-Inducible Expression of Nucleus- and Plastid Genome-Encoded Heat Shock Protein 70 Genes in the Marine Red Alga Neopyropia yezoensis
Source: Plants (Basel). 2023 May 23;12(11):2070. doi: 10.3390/plants12112070 (PMC10255470; doi:10.3390/plants12112070)
Supplement: Supplementary file 1 [file plants-12-02070-s001.zip › Figure S1.pdf]

```
CMP145C     ------------------------------------------------------------------------------------------------------------------------------------------------------------------------------------    0
KAA8492910  ------------------------------------------------------------------------------------------------------------------------------------------------------------------------------------    0
OSX76008    ------------------------------------------------------------------------------------------------------------------------------------------------------------------------------------    0
NyHSP70-1   ------------------------------------------------------------------------------------------------------------------------------------------------------------------------------------    0
AHC94268    ------------------------------------------------------------------------------------------------------------------------------------------------------------------------------------    0
OSX71346    ------------------------------------------------------------------------------------------------------------------------------------------------------------------------------------    0
NyHSP70-2   ------------------------------------------------------------------------------------------------------------------------------------------------------------------------------------    0
ALA56108    ------------------------------------------------------------------------------------------------------------------------------------------------------------------------------------    0
AHC94269    ------------------------------------------------------------------------------------------------------------------------------------------------------------------------------------    0
OSX74808    -------------------------------------------------------------------------------------------------------------------MSR------------DGSVVLRRSRRPS---GGRKLRRIAAAAVVAAAAALAMTTLVLRARGE--    48
NyBiP2      -------------------------------------------------------------------------------------------------------------------MMW------------GG-SVAPRRVRPR---GGRSLRRIVAAAAVAATAALAVTTLVSRTRGD--    47
ALA56111    -----------------------------------------------MNPPALTNACRILARR------------VSRRGRAARQTHLCCCSRARPCSRLS---------ASSRTMMG------------AG-SLAPRRVRPR---GGRSLRRIVAAAAVAATAALAVTTLVARARGE--    94
CMT579C     --------------------------------------------------------------------------------------------------------------------------------------MATRRF--------SYRRVSIWNWALVAVLVHVCCCLFGRAVLVGA    38
KAA8497180  -------------------------------------------------------------------------------------------------------------------MVE------------VPGRMAKGRVVPA---A---------------ATVLVCAALVC--SAV--    31
AHC94272    -------------------------------------------------------------------------------------------------------------------MMR------------A-GSLAPRRVRPC---GGRSLRRIVAAAAVAATVALAVTTLVARARGE--    47
NyBiP1      -------------------------------------------------------------------------------------------------------------------MAA------------T-PGSPARRPRPA---AAK--RRSVWTAVLVAAASIATATV--AVSAA--    43
OSX74071    -------------------------------------------------------------------------------------------------------------------MMG------------A-AGSPARRPRPA---A-P--RRSMWTAVLVAAASIAAAAV--SVSAA--    42
CMV163C     ------------------------------------------------------------------------------------------------------------------------------------------------------------------------------------    0
BAO23680    ------------------------------------------------------------------------------------------------------------------------------------------------------------------------------------    0
1802278A    ------------------------------------------------------------------------------------------------------------------------------------------------------------------------------------    0
NycpDnaK    ------------------------------------------------------------------------------------------------------------------------------------------------------------------------------------    0
AHC94270    ------------------------------------------------------------------------------------------------------------------------------------------------------------------------------------    0
ALA56110    -------------------------------------------------------------------------------------------------------------------------------------------------------MMRRFSSR-TAVS-------------SVP    15
KAA8491802  -----------------------------------------------------------------------------------------------------------------------------------------------MAGRGSSLVRTQWRNLVSCNA-------------RRG    24
CML205C     -------------------------------------------------------------------------------------------------------------------MLRFLLRAGARALHRAPALATLKVPEPVVELRQPSLLPQRR------------------------    41
NymtDnaK    MFLEGATLPLSTWLLGGRSSPLAVSLQALQRRRNLLRSTTSQPPRPCIDPRVVRRRCRWGTKTTWSVPLPVVGSLCGSPGHLPRPLTLLSCVHPTMASAVSARAALARVGACRPGLLGLAASGGGG----LAALRPAGRPTPSAAAAAAGAALRRW------------------------    152
ALA56109    -----------------------------------------------------------------------------------------------MASAVSARTALARVGSCRPGLLGLAAGGGGG----LAALRPAGHPAPSVVAAAAGAALRRW------------------------    57
AHC94271    ------------------------------------------------------------------------------------------------------------------------------------------------------------------------------------    0
```

HSP70 family signeture 1

```
CMP145C     ---------SKAKAIGIDLGTTYSCVAVMEGNKVEIIANEQGNRTTPSYVAFTE--TERLIGDAAKNQVALNPENTVFDAKRLIGRKFSDPTVQEDMKHWPFKVVQGPGDKPLIQVVAHG-DVKRFSPEEISAMVLTKMKDIAESYLGTKVTDAVITVPAYFNDSQRQATKDAGTIAGLN    168
KAA8492910  -------MAATGVAVGIDLGTTYSCVGVWQNDRVEIIANDQGNRTTPSYVAFTD--TERLIGDAAKNQVAMNPHNTVFDAKRMIGLRFDSPSVQADLKHFPFKVVGAEDNKPKIEVKYKG-ETKTFMPEEISAMVLGKMKEISEAYLAKEVKNAVVTVPARFNDSQRLATKDAGQIAGLN    170
OSX76008    --MADKKDVPSGPSIGIDLGTTYSCVGIWQNDRVEIIANDQGNRTTPSFVAFTD--SERLIGDAAKNQVAINAKNTVFDAKRMIGRKFSDPSVQSDMKHFSFKVVQKEGDKPVVEVLYKG-ETKQFSPEEISAMVLSKMREVAESYLGKEVKTAVVTVPAYFNDSQRLATKDAGTIAGLH    175
NyHSP70-1   --MADKKDVPSGPSIGIDLGTTYSCVGIWQNDRVEIIANDQGNRTTPSFVAFTD--SERLIGDSAKNQVAINATNTVFDAKRMIGRKFSDPSVQQDMKHFSFKVVQKEGDKPVVEVQYKG-ETKQFSPEEISAMVLSKMREVAESYLGKEVKTAVVTVPAYFNDSQRLATKDAGTIAGLH    175
AHC94268    --MADKKDVPSGPSIGIDLGTTYSCVGIWQNDRVEIIANDQGNRTTPSFVAFTD--SERLIGDAAKNQVAINATNTVFDAKRMIGRKFSDPSVQQDMKHFSFKVVQKEGDKPVVEVLYKG-ETKQFSPEEISAMVLSKMREVAESYLGKEVKTAVVTVPAYLNDSQRLATKDAGTIAGLH    175
OSX71346    ---------MGGPTIGIDLGTTYSCVGYWADDRVEIIANDQGNRTTPSFVAFTA--QERLVGDAAKNQAAVNAANTVYDVKRLIGRKMSEPSVQRDLKHFSYTVASKDG-KPFVDVEVKG-SRKQFSPEEISAMVLSKMREVAESYLGKEVKDAVVTVPAYFNDSQRQATKDAGLIAGLN    167
NyHSP70-2   --------MASGPAIGIDLGTTYSCVAYWADDRVEIIANDQGNRTTPSFVAFTT--TERLVGDAAKNQVAVNAANTVYDVKRLIGRHFSEPSVQRDLKHFSYPVVAKDGDKPFVQVEYKG-ATKQFSPEEISAMVLAKMREVAESYLGKEVKDAVVTVPAYFTDSQRQATKDAGVIAGLN    169
ALA56108    --------MASGPAIGIDLGTTYSCVGYWADDRVEIIANDQGNRTTPSFVAFTT--TERLVGDAAKNQVAVNAANTVFDVKRLIGRHFSESSVQRDMKHFSFAVVAKEGDKPFVQVEYKG-ATKQFSPEEISAMVLAKMREVAESYLGKEVKDAVVTVPAYFTDSQRQATKDAGVIAGLN    169
AHC94269    --------MASGPAIGIDLGTTYSCVGYWADDRVEIIANDQGNRTTPSFVAFTT--TERLVGDAAKNQVAVNAANTVFDVKRLIGRHFSESSVQRDMKHFSFAVVAKEGDKPFVQVEYKG-ATKQFSPEEISAMVLAKMREVAESYLGKEVKDAVVTVPAYFTDSQRQATKDAGVIAGLN    169
OSX74808    -TEVDLDEEDKGPVIGIDLGTTYSCVGVVENGEVTIIANDQGNRITPSYVAFSSNSNDRLIGDAAKNQAPMNPSNTVFDVKRFIGRRFDEPTVGKDRKMLPYAIIDKDN-KPQVEVSVNG-ASRMYTPEEISAMVLTRLKKTAEDYLGKRVSRAVITVPAYFSDAQRSATKDAGVIAGLD    225
NyBiP2      -TEVDLDEEDKGPVIGIDLGTTYSCVGVVENGEVTIIANDQGNRITPSYVSFSGDSTDRLIGDAAKNQAPLNPTNTVFDVKRFIGRRFDEPTVGKDRKMLPYAIVDKDN-KPQVEEAENG-ATKVYTPEEISAMVLTKLKKTAEDYLGKRVSRAVITVPAYFSDAQRSATKDAGVIAGLD    224
ALA56111    -SEVDLDEEDKGPVIGIDLGTTYSCVGVVENGEVTIIANDQGNRITPSYVSFSGDSTDRLIGDAAKNQAPLNPTNTVFDVKRFIGRRYDEPTVGKDRKMLPYAIVDKDN-KPQVEVAVNG-ATKMYAPEEISAMVLTKLKKTAEDYLGKRVSRAVITVPAYFSDAQRSATKDAGVIAGLD    271
CMT579C     KDASSGGGKIEGPVIGIDLGTTYSCVGVFKNGKVEIIANEQGNRITPSYVAFTD--KERLIGDAAKNQLALNPERTIFDVKRLIGRRFDEETVQKDIKLLPYKVVNKDG-KPYIRVEVRDGEVKTFSPEEISAMILGKMKKIAEDYLGKPVKNAVVTVPAYFNDAQRQATKDAGTIAGLT    215
KAA8497180  -HAAAEEKDKVGTVIGIDLGTTYSCVGVMRNGQVEIIANDQGNRITPSYVAFTP--EERLIGDAAKNQAAMNPQNTVFDVKRLIGRKFTDTTVQRDKKLLPYTIVNQGG-KPYISVDIKEGETKTFSPEEISAMVLGKMKLIAEEYLGEKVTHAVVTVPAYFNDAQRQATKDAGVIAGLT    207
AHC94272    -SEVDLDEEDKGPVIDIDLGTTYSCVGVVENGEVTIIANDQGNRITPSYVSFSGDSTDRLIGDAAKNQAPMNPSNTVFDVKRLIGRKFSDPTVQRDKKLLPFSIVNKDG-KPMVEVSVNK-AKKVMSPEEVSAMVLSKMKKTAEEYLGKEVTNAVVTVPAYFTDAQRQATKDAGQIAGLN    224
NyBiP1      --AADAEKPPVGTVIGIDLGTTYSCVGVMRNGHVEIIANDQGNRITPSYVAFTP--EERLIGDAAKNQAAMNPSNTVFDVKRLIGRKFSDPTVQRDKKLLPFSVVNKDG-KPMVEVSVNN-AKKVMSPEEVSAMVLTKMKKTAEEYLGKEVTNAVVTVPAYFTDAQRQATKDAGQIAGLN    217
OSX74071    -AAGDADKPPVGTVIGIDLGTTYSCVGVMRNGHVEIIANDQGNRITPSYVAFTP--EERLIGDAAKNQAAMNPSNTVFDVKRLIGRKFSDPTVQRDKKLLPFAIVNQDG-KPMVEVSVNS-DKKVMSPEEISAMVLTKMKKTAEEYLGKDVTNAVVTVPAYFTDAQRQATKDAGQIAGLN    217
CMV163C     ----------MAKVVGIDLGTTNSVIAVMEGGQPTVVPNSEGFRTTPSVVAYTKN-GDLLVGQIAKRQAVINPGNTFYSVKRFIGRKFSE--IEQEAKQVPYPVQADGKGNVRIFCSAKD---KFFAPEEISAQVLRKLVDSASQYLGEKVTQAVITVPAYFNDSQRQATKDAGKIAGLD    164
BAO23680    ----------MAKVVGIDLGTTNSVIAVMEGGKPTVIPNAEGFRTTPSVVAYTKN-GDRLVGQIAKRQAVINPENTFYSVKRFIGRKANE--VNEELKQVPYRVFDDPNGNVKIQCSTLD---KDFAPEEISAQVLRKLADDAGKYTGETIKQAVITVPAYFNDSQRQSTKNAGKIAGLD    164
1802278A    ----------MGKVVGIDLGTTNSVIAVMEGGKPTVIPNAEGFRTTASVVAYTKS-GDKLVGQIAR-QAVINPENTFYSVKRFIGRKQNE--ISQEIRQTSYNVKT-SGSSIKIECPALN---KDFAPEEISAQVLRKLVEDASTYLGETVTQAVITVPAYFNDSQRQATKDAGKIAGLD    162
NycpDnaK    ----------MGKVVGIDLGTTNSVIAVMEGGKPTVIPNAEGFRTTASVVAYTKS-GDKLVGQIARRQAVINPENTFYSVKRFIGRKQNE--ISQEIRQTSYNVKT-SGSSIKIACPALN---KDFAPEEISAQVLRKLVEDASTYLGETVTQAVITVPAYFNDSQRQATKDAGKIAGLD    163
AHC94270    ----------MGKVVGIDLGTTNSVIAVMEGGKPTVIPNAEGFRTTASVVAYTKS-GDKLVGQIARRQAVINPENTFYSVKRFIGRKQNE--ISQEIRQTSYNVKT-SGSSIKIACPALD---KDFAPEEISAQVLRKLVEDASTYLGETVTQAVITVPAYFNDSQRQATKDAGKIAGLD    163
ALA56110    FVRACSGGKVQGDVIGIDLGTTYSCVAIMEGDKPRVIENAEGFRTTPSVVAFKG--EEKLVGHSAKRQAVTNPSSTFYAVKRFIGRRFDDPATQKDIKAMPFKIVRHSNGDAWVE-NANG--KQFSPSQVGAFVLEKMKETAEAHLHKKVKNAVVTVPAYFNDAQRQATKDAGQISGLT    189
KAA8491802  LSTGAASDGAAGQVIGIDLGTTNSCVAVMQGKSARVIENAEGQRTTPSVVAFAPD-GEMLVGAPAKRQAITNSENTLFAIKRLIGHRFTDPETQKDVKTMPFKIVPADNGDAWVE--ANG--QKYSPSQIGAFVLSKMKKTAEDFLGQNVTNAVVTVPAYFNDAQRQATKDAGQIAGLN    198
CML205C     ---LRSTGAVQGDVVGIDLGTTNSCVAIMEGKAPRVIENAEGQRTTPSVVAFTSS-GERLVGIAAKRQAVTNPENTIFAAKRLIGRRYEDPEVQRDVKIMPYKIVRADNGDAWVE--AQG--NRYSPAQIGAFVLQKMKETAESFLGRTVNNAVITVPAYFNDAQRQATKDAGRIAGLN    212
NymtDnaK    ---KSSDAGIAGDVIGIDLGTTNSCVAVMQGSEARVIENAEGMRTTPSVVAFTAD-GEKLVGVPARRQAVTNPENTLFATKRLIGRRYDDEKTQEDIRQMPFKIVKGPNGDAWVS--AAG--EKYSPSQVGAFVLQKMRETAEAFLGRKVSSAVVTVPAYFNDSQRQATKDAGKIAGLN    323
ALA56109    ---KSSDAGIAGDVIGIDLGTTNSCVAVMQGSEPRVIENAEGMRTTPSVVAFSAD-GEKLVGVPARRQAVTNPENTLFATKRLIGRRYDDSKTQEDIRQMPFKIVKGPNGDAWVS--AGG--DNYSPSQVGAFVLQKMRETAEAFLGRKVSSAVVTVPAYFNDSQRQATKDAGKIAGLN    228
AHC94271    ----------------------------MQGSEPRVIENAEGMRTTPSVVAFSAD-GEKLVGVPARRQAVTNPENTLFATKRLIGRRYDDNKTQEDIRQMPFKIVKGPNGDAWVS--AGG--DNYSPSQVGAFVLQKMRETAEAFLGRKVSSAVVTVPAYFNDSQRQATKDAGKIAGLN    146
                                     .    :: * :* * * * *::      : *:*   *: *    *    *.:  **:** :  .      : :   : :       .  :           * ::.* :* ::    :   .     :. **:**** :.*:** :**:** *:**
```

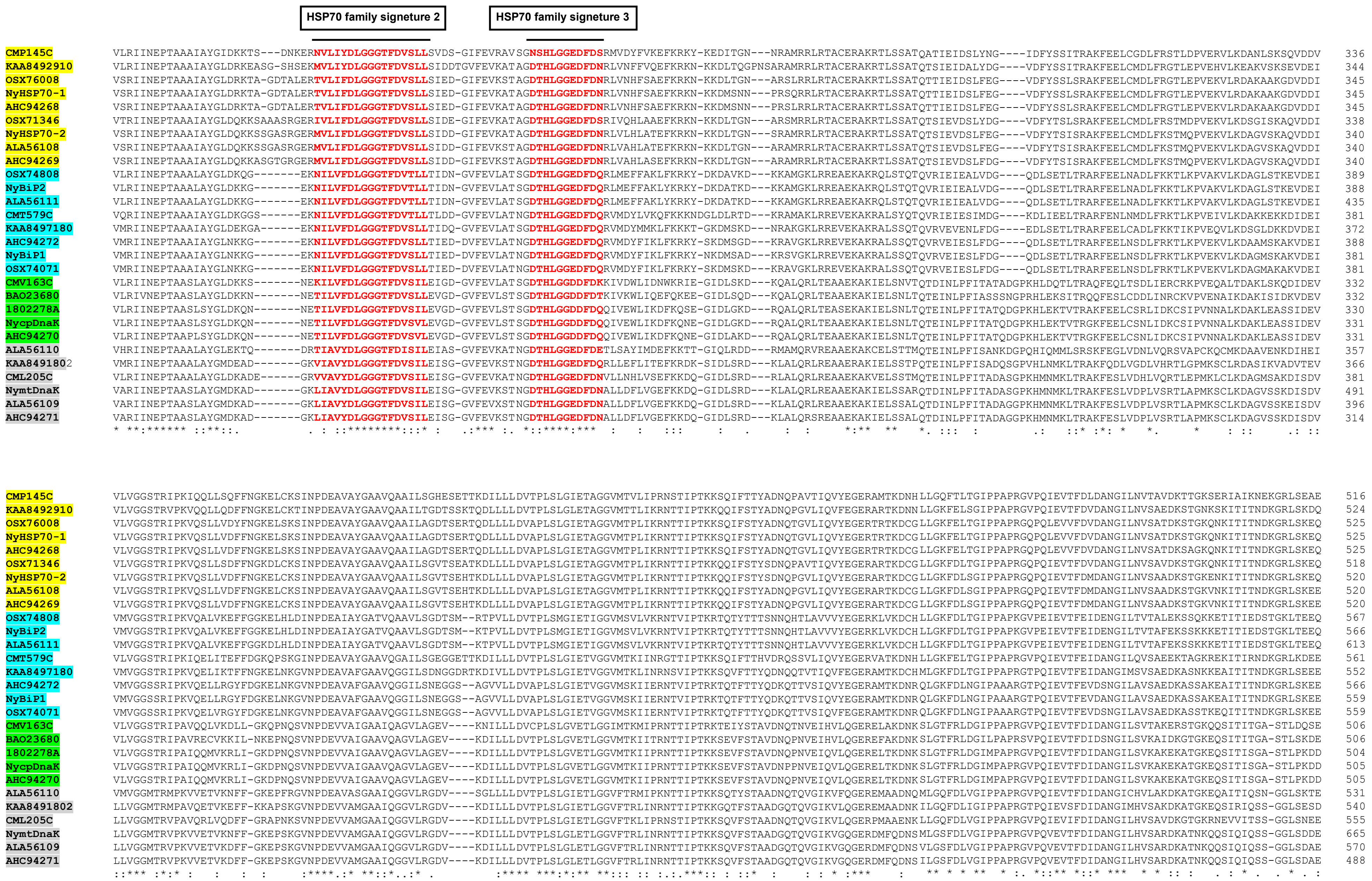

HSP70 family signature 2
HSP70 family signature 3
CMP145C 336
KAA8492910 344
OSX76008 345
NyHSP70-1 345
AHC94268 345
OSX71346 338
NyHSP70-2 340
ALA56108 340
AHC94269 340
OSX74808 389
NyBiP2 388
ALA56111 435
CMT579C 381
KAA8497180 372
AHC94272 388
NyBiP1 381
OSX74071 381
CMV163C 332
BAO23680 332
1802278A 330
NycpDnaK 331
AHC94270 331
ALA56110 357
KAA8491802 366
CML205C 381
NymtDnaK 491
ALA56109 396
AHC94271 314
CMP145C 516
KAA8492910 524
OSX76008 525
NyHSP70-1 525
AHC94268 525
OSX71346 518
NyHSP70-2 520
ALA56108 520
AHC94269 520
OSX74808 567
NyBiP2 566
ALA56111 613
CMT579C 561
KAA8497180 552
AHC94272 566
NyBiP1 559
OSX74071 559
CMV163C 506
BAO23680 506
1802278A 504
NycpDnaK 505
AHC94270 505
ALA56110 531
KAA8491802 540
CML205C 555
NymtDnaK 665
ALA56109 570
AHC94271 488

```
CMP145C        VERMVKEAEAMKAKDEEVRRTVEARNSLEQLAYSAKRTV-EEEQVAQSLSAGDKQKILDKTKEVLEWLEEN-GATASLDQIKNMQKELESVTMPIFTRMYQQAGGAADG--MPG---AGGMPG--------AGGMPG--AGGMPGAGGAGTG-------SGPTVEEVD   660
KAA8492910     IEKMVQDAEKYKAEDEIMKARVEAKNGLENYAYNVRNTI-QDEKIAGKLDADDKKAIEDKVEECLKWLEDN-SEA-STEEYTSMQKALEAVVNPIMTKMYQAGGGAPDMGGMPDMGGAGGAAGGTGG---------------------------------VQVDEVD   655
OSX76008       IEAMVQEAEKYKAEDEAAQAKIEARNGLENYCYNLKNTTVGDATVKEKLSEDDAKAIETIVADTLAWLEAN-DNA-EKEEYDAKLKEVEGVAHPIMTKLYAAG-G---------------APGGMPG-----------------AGGEAPMDTGGAEN-PDITVEDID   657
NyHSP70-1      IEAMVQEAEKYKAEDEAAQAKIEARNGLENYCYNLKNTTVGDATVKEKLSEDDAKAIETIVADTLAWLESN-DNA-EKEEYDAKLKEVEAVAHPIMTKMYAAGGG---------------APGGMPD-----------MS-GMGGAGAAPMDTGGADN-PDITVEDID   663
AHC94268       IEAMVQEAEKYKAEDEAAQAKIEARNGLENYCYNLKNTTVGDATVKEKLSEDDAKAIETIVADTLAWLESN-DNA-EKEEYDAKLKEVEAVAHPIMTKMYAAGGG---------------APGGMPD-----------MAGGMGGAGAAPMDTGGADN-PDITVEDID   664
OSX71346       VEAMVQEAEQFKAEDEAARERIEARNGLEAYLYNIKSSVLTDKAVKDKLSEDDQSTLERTITNALEWLESH-DDS-DKESYERKRKEVEEVVTPITARLHQAAGGGDGGGAGPMPGGMGGMPGGMGGMPGGMGGMPGGMPGGFPGAGGPA--PMD-TD-PNVTVEDID   680
NyHSP70-2      IERMVSEAESFKDEDEKARARIEARNSLEGYLYNVKNSTLSDKAVKDKLSEDDVGTLERTINNALEWLESH-DSS-EKEDYERKMSEVQEVVNPIMTRFHQSSGGGGAG----------GMPGGM------PGGMPGGMPGGMPGGGGAPMDTSG-SD-PNITVEDID   668
ALA56108       IERMVNEAESFKDEDEKARARIEARNGLEGYLYNVKNSTLADKAVKDKLSEEDASTLERTINNALEWLESH-DNS-DKEDYDRKQSEVQEVVNPIMTRFHQSTGGGGAG----------GMPGGM------P----GGMPGGMPGGGAAPMDTSG-AD-PNITVEDID   664
AHC94269       IERMVNEAESFKDEDEKARARIEARNGLEAYLYNVKNSTLSDKAVKDKLSEEDASTLERTINNALEWLESH-DMS-DKEDYERKQSEVQEVVNPIMTRFHQSTGGGGAG----------GMPGGM------PGGMPGGMPGGMPGGGAAPMDTSG-SD-PNITVEDID   668
OSX74808       IKKMVTEADSFADEDAEVSKKLNAKTDLDNYLSGLRVQLA--STYKDKLSRVDSRNIKEAVDEADSWLSGLNVEDVDIDDIKMHKREVEDVAGPVFTKLYAAGVDKEEL                                                                    674
NyBiP2         IKKMVKEADSFADEDAEVSKKLSAKTDLDNYLSGLRVQLT--STYKDKLSRVDSRNIKEAVDEADSWLSGLNIEEVDIDDIKMHKREVEDVAGPVFTKLYASGMDKEEL                                                                    673
ALA56111       IKKMVKEADSFADEDAEVSKKLSAKTDLDNYLSGLRVQLT--STYKDKLSRVDSRNIKEAVDEADSWLSGLNIEEVDIDDIKNHKREVEDVAGPVFTKLYASGIDKEEL                                                                    720
CMT579C        IQRMVREAEEYAEVDAKLKRKVDAKNNFENYIYQVRQMYEDKDK-KTKLSTDDIDKLKDSVESAQDWLDEH-GEASDAAAIEERMKAFQDVVQPIILKTYESAKGTGKDSSADSSADDDRDSEEHDEL                                                    687
KAA8497180     IERMVREAEEFAEEDAAFKKKVEQKNALENYIYSVRSQVEDEDKLADKISEDDKETIVNALKEAEDWLEEN-GADADGDDFETQLKDLQDIVAPIVSSLYGAGQG-GDEFGGDFEDDHEEL                                                           671
AHC94272       IERMVKEAEEFADADAEVKRKVEGKNSLETAIFSAKAAVEEGGSLVDKASEEDKESLKAAVKEAQDWLDES-GEDATADDLEGKLKDLQEIIQPIMAKGGAAGGGSGAAQGGEEDDSKEEL                                                           686
NyBiP1         IERMVKEAEEFADADAEVKRKVEGKNSLETAIFSAKAAVEEGGSLVDKASEEDKESLKAAVKEAQEWLDES-GEDATADDLEAKLKDLQEIVQPIMAKGGAAGGGSGAAQGGEEDDTKEEL                                                           679
OSX74071       IERMVKEAEEFADQDAEVKRKVEGKNSLETAIFAAKSAVEEGGSLADKASDEDKESLKAAVKEAQDWIEES-ADDATADDLEAKLKDLNEIVQPIMAKGGAAGGG-GAPPGGEEDDTKEEL                                                           678
CMV163C        IERMVKEAEKNAEEDRKKREQIETKNLAESVYYQAEKMG---LK--------------DNAQELKNAIDQL---DY--EGMKNLTQQVQTLIAQKAS-----ETSNAKTNGKASEK------------------------EDVIDADFKAQE                          607
BAO23680       VDSMVNTAQQYEAEDKIKREQIDIKNQADSLCYQSEKQL---KELSDKITEDDKNKLESLIKQLRDSVKEE---NF--DSMKALSKELEQELMAIGQKIYSQKNESNSPNSNPSEN------------------------DNVIDTEGN                             623
1802278A       VERMVKEAEENFDVDQKRRKNIDIRNQAESLCYQSEKQV---KEFEDKIDEELKNRITNLISELRSNLEKE---EL--DSIEANSEKLQNALMEIGKNATSAEKDT----QNASND------------------------DTVIDTDFSEAK                          620
NycpDnaK       VERMVKEAEENFDTDQKRRKNIDTKNQAESLCYQAEKQV---KEFEDKISQDLKIKIEELITELRSSLEKE---EY--DNIESISQQLQNALMDIGKNAAQTESKD----TKAKDD------------------------DTVIDTDFSEAK                          621
AHC94270       VERMVKEAEENFDTDQKRRKNIDTKNQAESLCYQAEKQI---KEFEDKISQDLKTKIEELITELRSSLEKE---EY--ENIESISQRLQNSLMDIGKMAAQAESKN----TNTKDD------------------------GTVIDTDFSEAK                          621
ALA56110       IERMIREGEQHADNDRKKREIVETKNNAETQLNTAEKQL---GEWKYVSDAEKAN-VRELLAALKKEMENP---NSSKDDLAGATDKLQKAVMECGRTEYQQAAAQNQSSGAQQG-------------------------GDQQQQQQQQQQSGEQKPEGEQK            663
KAA8491802     IQEMVRNAESYAEEDKKRREKVEHRNTLENLVYQAEKSL---KDYRDKLNAEQIEKIEVAISEARDASKSE---TMSGEEMKEKITQLEQAVMIIGANMYKNPGNVDKEASAS--------------------AQAGAQKGGAQDAEFMDAEDMSDKKKDESKN               678
CML205C        IEKMIKDAEMHAEEDRRRQAAVEAKNEADSLLYTTERTL---SEHRAKLSATDVETVEKAAQDLRAVLEKD---ATAADTIREKTKVLQQAAMRIGEAIYRASQASQSTQQAQQSQ------------------------SETPEAEFKDVNQDSDEKK----QQGKGGS         691
NymtDnaK       VSEMVKRAEAYAKEDKARREAIEAKNDADSLLYSTEKTL---SEHREKMPADDVGAVESAMTALRSELEKE---EPSTKELKDLTDALQKASMKVGEAMYKASQAANASPSDGGADTAGGGAAGEDGTKAEGAKTDGANADGAQEAEFRDADADSDGAKDAKDKKASGGSSA   831
ALA56109       VSEMVKRAEAYAQEDKQRREGIEAKNDADSLLYSTEKTL---SEHREKMPADDVAAVESAMTALRAEVEKE---DPSTKELKDLTDALQKASMKVGEAMYKASQAANASPSDGSADAADAGAAGESGSKAENTKAGGAKADGAQEAEFRDADADSDGAKDAKDKKASGGGSA   736
AHC94271       VSEMVKRAEAYAQEDKQRREGIEAKNDADSLLYSTEKTL---SEHREKMPADDVAAVESAMTALRAEVEKE---DPSTKELKDLTDALQKASMKVGEAMYKASQAANASPSDGGSGAADAGAAGEGGSKADGAKAGGAKADGAQEAEFRDADADSEGVKDSKDKKASGGGSA   654
               :. *:  .:     *      :. :.  :      .                          .                .:
```

Figure S1. The presence of subcellular localization signature motifs in four classes of HSP70 proteins from the Bangiales and unicellular red algae. The amino acid sequences of 28 HSP70 proteins were aligned using the Clustal Omega tool (https://www.ebi.ac.uk/Tools/msa/). According to the phylogenetic classification shown in Fig. 1, the accession numbers and gene names of the cytoplasmic, ER, CP, and MT HSP70s are highlighted with yellow, blue, green, and gray backgrounds, respectively. The three HSP70 family signatures [54] are indicated in red font. Amino acid sequence motifs specifically found in each class are also highlighted with backgrounds identical to the color of their corresponding classes. The asterisks, colons, and periods represent identical amino acids, amino acids of stronger similarity groups, and amino acids of weaker similarity groups, respectively.
