# Supplementary material for: Membrane Fluidization Governs the Coordinated Heat-Inducible Expression of Nucleus- and Plastid Genome-Encoded Heat Shock Protein 70 Genes in the Marine Red Alga Neopyropia yezoensis
Source: Plants (Basel). 2023 May 23;12(11):2070. doi: 10.3390/plants12112070 (PMC10255470; doi:10.3390/plants12112070)
Supplement: Supplementary file 1 [file plants-12-02070-s001.zip › Figure S2.pdf]

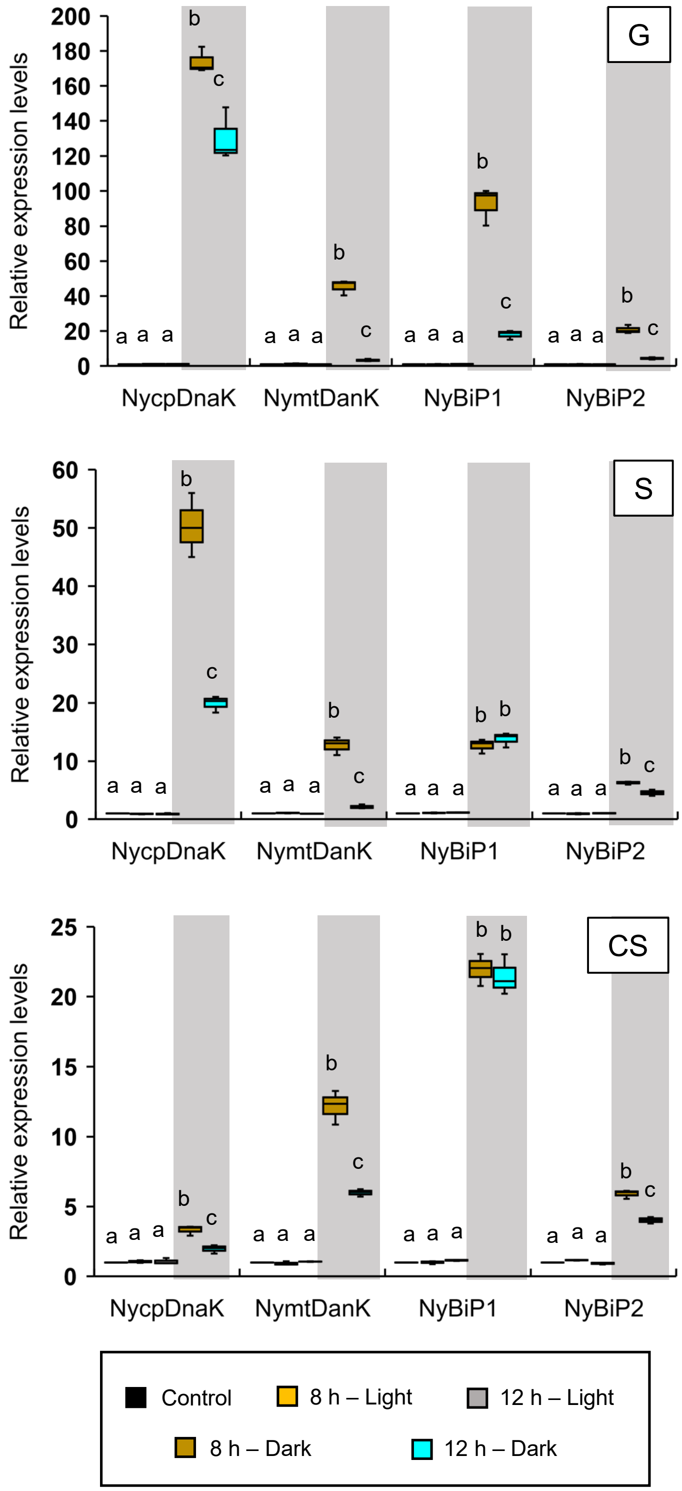

Figure S2. Requirement of dark treatment for the second expression peak of genes for ER and organellar HSP70 in the three life stages of *Neopyropia yezoensis* at 25° C. The second expression peaks at 8 and 12 h into heat stress exposure were compared under light and darkness (indicated by shading). Values on the y-axis represent the fold-change of the expression of each gene relative to that at 0 h. Significant differences in the expression level in the three life stages, indicated by different letters, were defined from triplicate independent replicate data using a one-way ANOVA with a Tukey's test ($p < 0.05$). G, gametophyte; S, sporophyte; CS, conchosporophyte.
