# Supplementary material for: Membrane Fluidization Governs the Coordinated Heat-Inducible Expression of Nucleus- and Plastid Genome-Encoded Heat Shock Protein 70 Genes in the Marine Red Alga Neopyropia yezoensis
Source: Plants (Basel). 2023 May 23;12(11):2070. doi: 10.3390/plants12112070 (PMC10255470; doi:10.3390/plants12112070)
Supplement: Supplementary file 1 [file plants-12-02070-s001.zip › Figure S3.pdf]

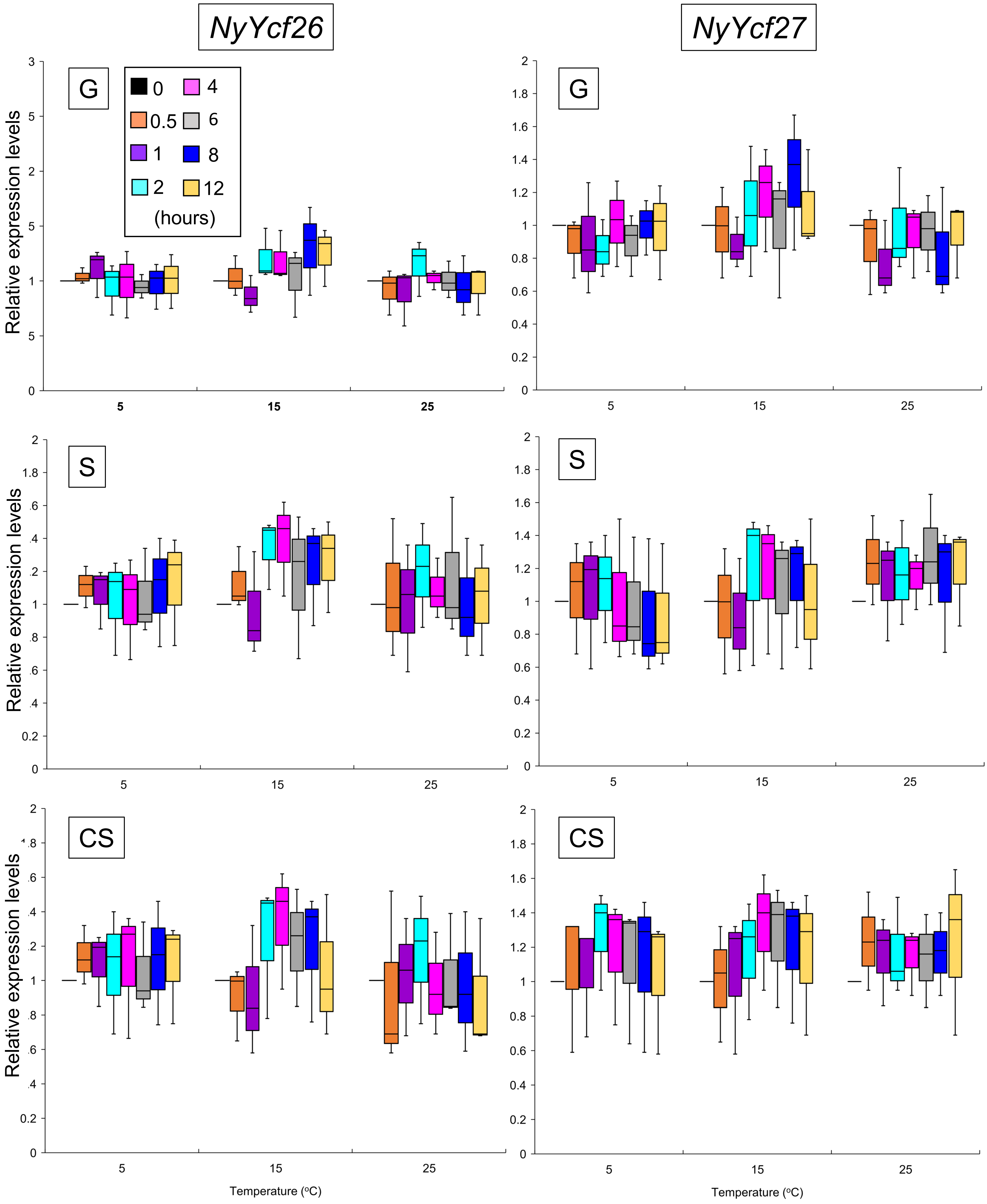

Figure S3. Effects of tMemperature changes in expression of *NyYcf26* and *NyYcf27* in three life cycle generations of *Neopyropia yezoensis*. Values on the Y axis represent the fold change of relative quantification of each gene, whose significant differences, significant differences in expression level in the three life cycle generations, indicated by different letters in the box ploys, were defined from triplicate independent replicates by Tukey's test ($p < 0.05$) in one-way ANOVA. G, gametophyte; S, sporophyte; CS, conchosporophyte.
