# Supplementary material for: Membrane Fluidization Governs the Coordinated Heat-Inducible Expression of Nucleus- and Plastid Genome-Encoded Heat Shock Protein 70 Genes in the Marine Red Alga Neopyropia yezoensis
Source: Plants (Basel). 2023 May 23;12(11):2070. doi: 10.3390/plants12112070 (PMC10255470; doi:10.3390/plants12112070)
Supplement: Supplementary file 1 [file plants-12-02070-s001.zip › Table S1.pdf]

Table S1. Sequences of primer sets used for quantitative real time PCR analyses.

**Primer name**	**Primer sequence (5' - 3')**	**Annealing temperature (ºC)**	**Product size (bp)**
Q-NyBiP1-F	ACGCTTAAGCCTGTGGAGAA	60	148
Q-NyBiP1-R	CCTTGTTCAGCTCCTTACCG		
Q-NyBiP2-F	GAGCTGCACTTGGACATCAA	59	108
Q-NyBiP2-R	ATCAAGCAGAACTGGCGTCT		
Q-NycpDnaK-F	CTATTGGTGCAGCCGTACAAGC	58	107
Q-NycpDnaK-R	CACGCCACCCAGAGTTTCC		
Q-NymtDnaK-F	CGGCGGAGAAGGCTAAGATTG	59	114
Q-NymtDnaK-R	CGCGGGTCAGCTTCATGTTC		
Q-NyYcf26-F	TCTTTAATGCCGAAGGGCAAA	58	100
Q-NyYcf26-R	TCGCTGCGTAAACATTCGTA		
Q-NyYcf27-F	GCGTTAGGTGAAGTCTGCGA	60	100
Q-NyYcf27-R	GAATGCGAGCTTCTAGCTCCT		
Q-NyeIF4A-F	GCTTTCTGTCTGGACGAGG	59	181
Q-NyeIF4A-R	TCTTCACAAGGATGCGGAT		
